# BCL-2 and Bax Expression in Skin Flaps Treated with Finasteride or Azelaic Acid 

**Published:** 2012

**Authors:** Seyyed Abdulmajid Ayatollahi, Marjan Ajami, Hamed Reyhanfard, Yasin Asadi, Mansour Nassiri-Kashani, Mehdi Rashighi Firoozabadi, Sayed Hossein Davoodi, Esmaeil Habibi, Hamidreza Pazoki-Toroudi

**Affiliations:** a *Department of pharmacognosy, School of Pharmacy, Shahid Beheshti University, M.C., Tehran, Iran. *; b *School of Pharmacy and Phytochemistry Research Centre, Shahid Beheshti University of Medical Sciences, Tehran, Iran. *; c *Faculty of Nutrition Sciences and Food Technology, Shahid Beheshti University of Medical Sciences and Health Services. *; d *Physiology Research Center, Tehran University of Medical Sciences, Tehran, Iran.*; e *Center for Research and Training in Skin Diseases and Leprosy, Tehran University of Medical Sciences, Tehran, Iran. *; f *Faculty of Library and Information Science, Islamic Azad University, Tehran Science and Research Unit, Tehran, Iran. *; g *Nano Vichar Pharmaceutical Ltd, Tehran, Iran. *

**Keywords:** Azelaic acid, Apoptosis, Finasteride, Skin flaps

## Abstract

Despite all modern surgical techniques, skin flap that is considered as the main method in most reconstructive surgeries puts the skin tissue at danger of necrosis and apoptosis derived from ischemia. Therefore, finding a treatment for decreasing the apoptosis derived from flap ischemia will be useful in clinic.

In present study, we evaluated the effect of azelaic acid 20% and finasteride on expression of BCL-2 and bax proteins after the skin flap surgery. For this purpose, 21 rats were entered in three groups including control, azelaic acid 20% and finasteride, all experienced skin flap surgery and then flap tissue was assessed for determining the expression of proteins in 5 slices prepared from each rat that were graded between – to +++ scales.

Both azelaic acid and finasteride increased the expression of BCL-2 protein (p < 0.05) and decrease the expression of bax protein (p < 0.05).

These results suggested an antiapoptotic role for finasteride and azelaic acid in preserving the flap after the ischemia reperfusion insult.

## Introduction

Skin flap is considered as the main method in most reconstructive surgeries (1) and its application in providing suitable tissue coverage has an essential role in increasing the patients’ life quality (2). Despite the rapid progresses in skin flap application in clinic, ischemia may restrict its utility (3). The phenomenon of ischemia-reperfusion (IR) has been postulated to be an important event in skin flap surgery which leads to major complications and loss of tissue (4) but also involves in other chronic wounds such as venous stasis and diabetic foot ulcers (5-6). Therefore, finding the treatments which reduce flap injury would be highly advantageous in reconstructive surgery. 

Oxidative stress derived from IR injury through producing the reactive oxygen species (ROS) and oxidative damage causes DNA modification, lipid peroxidation and secretion of inflammatory cytokines (7-8). In addition, apoptosis is induced with ischemic insults in skin tissue (8).

Plenty of proteins and number of genes regulate the apoptosis, in which two pairs of proteins are more important (including BCL-2 and Bax). BCL-2 family of proteins is found in external surface of mitochondria and divided to 3 groups including: antiapoptotic proteins like BCL-2 and BclxL, proapoptotic proteins like Bax and BAD and the proteins with apoptotic activity like Bik (9-11). After occurring a severe damage in the DNA of cells in a way that cannot be repaired, it will undergo the apoptosis in which activation of p53 causes Bax (BCL-2 associated x-protein). Bax protein exists in the external membrane of mitochondria with some other proteins making the complex of Apoptosome, which has the key role in activating the caspases and inducting the apoptosis (12-13).

Yet, it has been shown that various pharmacological agents including inducible nitric oxide synthase (iNOS) (14), botulinum toxin A (15), Hemoglobin vesicles (16), Erythropoitin (17), University of Wisconsin solution (18) and Enalapril (19) protects against the ischemia-induced injury of skin flaps. In present study, we evaluated the effects of two different drugs, finasteride and Azelaic acid on BCL-2 and bax expression after the skin ischemia reperfusion.

## Experimental

This experiment received the approval of ethical committee of the Center for Research and Training in Skin Disease and Leprosy, Tehran University of Medical Sciences. Twenty-one Sprauge-Dawley male rats (200-250 g) were chosen for random pattern cranial-based skin flap elevation (19). The rats were divided into 3 groups of 7 rats including one control and two treatment groups, in flap tissue of which the protein expression was evaluated through receiving different pharmacologic agents for preconditioning the flaps. All along the study period, the rats were situated in single cages supplied with ample water and food. Preoperative preparations included shaving, administrating (ROS) and oxidative damage causes DNA modification, lipid peroxidation and secretion of inflammatory cytokines (7-8). In addition, apoptosis is induced with ischemic insults in skin tissue (8).

**Table 1:** Amount of immunostaining for BCL-2 protein in skin flap specimens in three different groups.

**Groups**	**BCL-2 expression**					
	**-**	**+/-**	**+**	**++**	**+++**	**Total**
**Control**	12 (34.3%)	13 (37.1%)	8 (22.9%)	2 (5.7%)	0 (.0%)	35 (100.0%)
Azelaic acid*	7 (20.0%)	10 (28.6%)	12 (34.3%)	5 (14.3%)	1 (2.9%)	35 (100.0%)
Finasteride*	7 (20.0%)	7 (20.0%)	14 (40.0%)	6 (17.1%)	1 (2.9%)	35 (100.0%)

*p < 0.05 compared to the control group

Plenty of proteins and number of genes regulate the apoptosis, in which two pairs of proteins are more important (including BCL-2 and Bax). BCL-2 family of proteins is found in external surface of mitochondria and divided to 3 groups including: antiapoptotic proteins like BCL-2 and BclxL, proapoptotic proteins like Bax and BAD and the proteins with apoptotic activity like Bik (9-11). After occurring a severe damage in the DNA of cells in a way that cannot be repaired, it will undergo the apoptosis in which activation of p53 causes Bax (BCL-2 associated x-protein). Bax protein exists in the external membrane of mitochondria with some other proteins making the complex of Apoptosome, which has the key role in activating the caspases and inducting the apoptosis (12-13).

Yet, it has been shown that various pharmacological agents including inducible nitric oxide synthase (iNOS) (14), botulinum toxin A (15), Hemoglobin vesicles (16), Erythropoitin (17), University of Wisconsin solution (18) and Enalapril (19) protects against the ischemia-induced injury of skin flaps. In present study, we evaluated the effects of two different drugs, finasteride and Azelaic acid on BCL-2 and bax expression after the skin ischemia reperfusion.

This experiment received the approval of ethical committee of the Center for Research and Training in Skin Disease and Leprosy, Tehran University of Medical Sciences. Twenty-one Sprauge-Dawley male rats (200-250 g) were chosen for random pattern cranial-based skin flap elevation (19). The rats were divided into 3 groups of 7 rats including one control and two treatment groups, in flap tissue of which the protein expression was evaluated through receiving different pharmacologic agents for preconditioning the flaps. All along the study period, the rats were situated in single cages supplied with ample water and food. Preoperative preparations included shaving, administrating artificial tears and scrubbing with isopropyl alcohol and povidone-iodine. Intraperitoneal administration of a Ketamine-Xylazine combination (50 and 10 mg/Kg respectively, Pake-Davis Pharmaceutical Co., Cambridge, UK) was performed for the induction of general anesthesia.

Dorsal surface of rat body was used for taking flap. Before the surgery, 1 mL of each pharmacological agent under study was injected subcutaneously at spots 5.5, 6.5 and 7.5 cm distant from caudal margin of flap. In control group, normal saline was merely used. In two experimental groups, the rats were injected azelaic acid (100 mg/flap, Merck) or (5 mg/Kg, Reddy India).

Flap elevation was performed through making two parallel caudal to cephalad incisions extending from inferior angle of scapulae to superior border of pelvic bones with distance of 3 cm from each other on the mice backs and as a result, 8 × 3 cm flaps were created after the final incision connecting distal ends of two parallel cuts and leaving a 3-cm-base connected to body for blood supply. After raising the flap and detaching it from underlying fascia, an impermeable plastic barrier, similar in size to the flap (8 × 3 cm), was interposed between the flap and its corresponding bed (20).

On the 7th post operative day, tissue samples from flap area were prepared for the measurement of apoptotic and anti apoptotic proteins in the tissue (21).

*Immunohistochemical analysis*

Tissue samples were put in PBS 0.01 M and preserved in -70ºC and were studied through alkaline phosphatase immunohistochemistry. Five micron-thick sections were obtained via microtome and transferred onto adhesive slides. The sections were kept in the autoclave at 37°C for 16 h and at 60°C for 20 min. Then, they were deparaffinized and dehydrated with immersion into xylene twice for 10 min and into alcohol twice for 2 min. Later, the specimens were incubated in 3% H2O2 for 5 min to inhibit the activation of endogenous peroxidases. After the reaction with primary antibody, the reaction of secondary labeled antibodies (Rabbit polyclonal to BCL-2; phospho S87, ab73985, abcam and Rabbit polyclonal to Bax; ab69643, abcam) with alkaline phosphatase were applied for immunohistochemical analysis of BCL-2 and bax, respectively (dilution 1.100). In immunohistochemistry BCL-2 or Bax, proteins take the dye and this shows the amount of protein expressed by cells quantitatively (22-23).

Mayer›s hematoxylin was used as the counterstain and slides were examined through light microscopy. The results of the immunostaining were analyzed semiquantitatively. The percentage of positive keratinocytes (the amount of staining) was recorded as follows: (–) no expression; (+/–) immunostaining in occasional cells (weak expression); (+) immunostaining in 1–25% of cells (mild expression); (++) immunostaining in 26-50% of cells (strong expression); and (+++) immunostaining in > 50% of cells (very strong expression) (24-25).

*Statistical analysis*

The results of the study were statistically analyzed using SPSS 14 program (Windows, Microsoft, USA). Mann-Whitney›s U*-*test was utilized for the comparison of numerical data. A p-value of α ≤ 0.05 was considered as significant.

## Results and Discussion

The results of immunohistochemical analysis of BCL-2 and bax are shown in Tables 1 and 2. In the control group, the expression of BCL-2 was low and approximately in 71% of slides, the level of BCL-2 expression was less than mild (+). However, after 7 days, the expression of bax in the control group of skin flap had been increased, while in more than 68.6% of slices, the expression was more than weak grade. The results for azelaic acid and finasteride were converse, and the expression of BCL-2 was increased in these groups to more than weak (+/-) in approximately 51.5% and 60% of them, respectively (p < 0.05). On the other hand, the expression of bax protein was significantly decreased via the treatment with azelaic acid and finasteride and reached less than mild grade in approximately 60% and 51.4% of them, respectively (p < 0.05).

**Table 2:** Amount of immunostaining for bax protein in skin flap specimens in three different groups.

**Groups**	**Bax expression**					
	**-**	**+/-**	**+**	**++**	**+++**	**Total**
**Control**	3 (8.6%)	8 (22.9%)	11 (31.4%)	10 (28.6%)	3 (8.6%)	35 (100.0%)
Azelaic acid*	7 (20.0%)	14 (40.0%)	7 (20.0%)	7 (20.0%)	0 (.0%)	35 (100.0%)
Finasteride*	6 (17.1%)	12 (34.3%)	12 (34.3%)	4 (11.4%)	1 (2.9%)	35 (100.0%)

In present study, treatment with azelaic acid and finasteride before the skin flap procedure produced a significant and different profile of apoptotic protein expression after 7 days, in which the ratio of BCL-2 to bax has been increased, compared to the control group.

In our previous study, we evaluated the effects of finasteride or azelaic acid 20% on skin flap viability and showed that each of these two treatments are able to significantly reduce the necrotic area of skin flap (26). In addition to the antinecrotic properties of azelaic acid and finasteride, results of present study confirmed the protective effects of azelaic acid and finasteride against the ischemia/reperfusion (I/R) injury by means of changing the protein expression in which led to the decreased apoptosis rate after skin flap procedure.

In present work, the effects of finasteride and azelaic acid on increasing BCL-2 and decreasing the bax expression in skin flaps have some controversies with studies on the prostate cancer cells. The effect of finasteride on prostate tissue in patients undergoing radical prostatectomy, showed decreased apoptotic factors, caspase-7 and IGFBP-3 in cancer cells, while having little to no effect on caspase-3, insulin growth factor-1, BCL-2, p53 and p21 (27). However, in an *in-vitro *model for prostate cancer, finasteride caused apoptosis and increased immunoreactivity for pro-apoptotic Bax whereas decreased antiapoptotic BCL-2 and BCL-XL expression (28). Treatment of rats with finasteride led to a slight decrease in Bax and a significant reduction in BCL-2 expression was observed at dose of 100 mg/Kg body weight (29). In the case of azelaic acid, there are also studies that have shown converse results; for example, it has been shown that the cardioprotective and antiapoptotic effects of 17Beta-estradiol were blocked with azelaic acid, as a thioredoxin (Trx) reductase inhibitor, since 17Beta-estradiol acts via the activation of Trx reductase (30) that regulates the levels of intracellular ROS and modulates the intracellular oxidative states, which may be important for the cellular function, survival, and death (31).

It should be mentioned that prostatic hyperplasia demonstrates increasing in BCL-2 protein but no change in the expression of Bax, BCL-X, and Bak (32). Therefore, the interaction of finasteride or azelaic acid with these cells could be different from the skin tissue after ischemia. Moreover, there are some findings that confirm the results of present study. Finasteride primarily, was marketed for the treatment of benign prostatic hypertrophy (33) and this was derived from its inhibitory effects on 5-alpha-reductase activity (34). In addition, azelaic acid has shown a potent inhibitory effect on 5-alpha-reductase activity and this effect was detectable at concentrations as low as 0.2 mmol/L and was completed at 3 mmol/L (35). Enzyme 5-alpha-reductase is involved in the catalyzing of testosterone to dihydrotestosterone (DHT) conversion (36). It has been shown that the inhibition of dihydrotestosterone (DHT) increases the expression of iNOS in testis and epididymis of rats (37). In our previous study, the administration of L-NAME prior to the finasteride and azelaic acid blocked its effects on reducing the flap necrotic area that suggested involvement of NO in this pathway. Therefore, we suggested that finasteride and azelaic acid has eliminated the inhibitory effects of DHT on iNOS expression through inhibiting 5-alpha-reductase activity and decreasing testosterone to dihydrotestosterone (DHT) conversion and consequently, this Nitric Oxide-Dependent pathway (NO-dependent pathway) of IPC has been triggered.

Cytotoxic effects of nitric oxide (NO) derived from inducible nitric oxide synthase (iNOS), are considered to be one of the major causes of inflammatory diseases. On the other hand, protective effects of NO on toxic insult-induced cellular damage/apoptosis have been demonstrated recently. In the study by Yamaoka J *et al*., it has been demonstrated that NO from NO donor suppressed UVB-induced apoptosis of murine keratinocytes. In addition, NO significantly suppressed the activities of caspase 3, caspase 8 and caspase 9 which had been upregulated through UVB radiation. NO also suppressed p53 expression that had been upregulated through UVB radiation and upregulated BCL-2 expression that had been down-regulated by UVB radiation. These findings suggested that NO might suppress UVB-induced keratinocyte apoptosis through regulating the apoptotic signaling cascades in p53, BCL-2, caspase3, caspase8 and caspase9 (38). In fact, ultraviolet (UV) irradiation of human skin leads to inducible nitric-oxide synthase (iNOS) expression in keratinocytes and endothelial cells (ECs). Searching for the molecular mechanism responsible for the protective effect, Suschek CV *et al*. found that protecting the UVA-induced apoptosis is tightly correlated with NO-mediated increases in BCL-2 expression and a concomitant inhibition of UVA-induced overexpression of Bax protein. NO, either endogenously produced or exogenously applied and the iNOS-derived NO, fully protects against the UVA-induced cell damage and death via the modulation of BCL- 2 family proteins (39). Therefore, it is possible for azelaic acid and finasteride to modulate apoptotic proteins after the ischemia reperfusion through regulating NO and related pathways.

In conclusion, the results of present study suggested an antiapoptotic role for finasteride and azelaic acid in skin flap model through increasing the BCL-2 expression and decreasing the bax expression. However, the precise mechanism and involved pathways remained to be determined.
